# Prognostic value of late gadolinium enhancement cardiac MRI for ICD therapy in non-ischaemic cardiomyopathy: A 5-year cohort study

**DOI:** 10.1007/s12471-025-01946-3

**Published:** 2025-03-25

**Authors:** Luuk H. G. A. Hopman, Marthe A. J. Becker, Sanna H. M. de Haas, Anne-Lotte C. J. van der Lingen, Mischa T. Rijnierse, Pranav Bhagirath, Michiel J. J. M. Zumbrink, Louise R. A. Olde Nordkamp, Lourens F. H. J. Robbers, Marco J. W. Götte, Vokko P. van Halm, Cornelis P. Allaart

**Affiliations:** 1 Department of Cardiology, Amsterdam University Medical Centre, Amsterdam, The Netherlands; 2 Department of Cardiology, Jeroen Bosch Hospital, Den Bosch, The Netherlands

**Keywords:** Non-ischaemic cardiomyopathy, Implantable cardioverter-defibrillators, Magnetic resonance imaging, Late gadolinium enhancement, Risk factors, Survival analysis, Electrophysiology, Retrospective studies

## Abstract

**Aim:**

To evaluate the impact of the 2023 Dutch national guidelines for primary prevention implantable cardioverter-defibrillator (ICD) implantation on outcomes in non-ischaemic cardiomyopathy (NICM) patients and to assess the role of late gadolinium enhancement cardiac magnetic resonance imaging (LGE-CMR) in predicting ICD therapy.

**Methods:**

This retrospective, single-centre observational exploratory cohort study included patients with NICM who received a primary prevention single-chamber, dual-chamber or subcutaneous ICD between January 2008 and April 2022 and underwent LGE-CMR prior to implantation. Patients were classified into LGE+ and LGE− groups based on the presence of late enhancement detected by CMR. The primary endpoint was time to first appropriate ICD therapy. The secondary endpoint was all-cause mortality.

**Results:**

Of the 258 NICM patients in the database, a total of 85 patients were included, of whom 41 had LGE on CMR. After a 5-year follow-up period, appropriate ICD therapy occurred in 20% of the patients in the LGE+ group and 14% of patients in the LGE− group (*p* = 0.37). All-cause mortality was 7% in the LGE+ group and 14% in the LGE− group (*p* = 0.46). Multivariable analysis showed no parameters significantly associated with appropriate ICD therapy.

**Conclusion:**

Applying the 2023 national guidelines retrospectively on a population of NICM patients with a primary prevention ICD indication demonstrated no significant association between LGE on CMR and appropriate ICD therapy over a follow-up period of 5 years. These findings underscore the need for further research and randomised trials to refine risk stratification and ICD implantation guidelines in NICM, ideally leveraging a multicentre approach to address current limitations in sample size and enhance the generalisability of the results.

**Supplementary Information:**

The online version of this article (10.1007/s12471-025-01946-3) contains supplementary material, which is available to authorized users.

## What’s new?

- Under stringent Dutch guidelines requiring LGE for ICD implantation, only a portion of NICM patients undergoing CMR met the criteria, highlighting a significant shift in clinical practice.
- This study challenges the predictive value of LGE for ICD therapy in NICM patients, as no significant differences in appropriate ICD therapy or mortality were found between LGE+ and LGE− groups.
- Findings underscore the need for large-scale, multicentre studies to validate the role of LGE and explore additional risk stratification tools in NICM patients.

## Introduction

In recent years, controversy has intensified regarding the necessity of primary prevention implantable cardioverter defibrillator (ICD) implantation in patients with heart failure and a reduced ejection fraction (HFrEF) due to non-ischaemic cardiomyopathy (NICM) [1]. Current international guidelines recommend determining ICD implantation eligibility based on risk assessment, considering left ventricular ejection fraction (LVEF), optimal medical therapy (OMT), and an expected survival of more than 1 year with a good functional status [2]. These guidelines are supported by meta-analyses indicating a survival benefit favouring primary prevention ICD therapy when applying these criteria. However, the benefits of cardiac resynchronisation therapy (CRT) defibrillators (CRT-D) may confound these results [1, 3].

Advancements in medical therapy over the past decades, particularly in heart failure treatment, including optimised medical management, have led to significant improvements in both the risk of sudden cardiac death (SCD) and life expectancy. These changes may impact the clinical benefit of prophylactic ICD implantation in NICM patients [4]. Moreover, the majority of patients who receive an ICD never experience appropriate therapy, and the incidence of inappropriate shocks is equal to or even exceeds that of appropriate shocks [1, 5, 6]. Furthermore, complications associated with ICD implantation, including infection, lead dislodgement and device malfunction, raise additional concerns. Consequently, the clinical and cost effectiveness of the current NICM ICD strategy appears suboptimal, suggesting that re-evaluation of the current guidelines is inevitable.

Clinical trials such as the CMR-ICD-DZHK23 Trial (NCT04558723), BRITISH (NCT05568069), and SPANISH‑1 (NCT06055504) are ongoing and will re-evaluate the appropriateness of the current ICD strategy in this specific patient population [7]. Importantly, these studies focus primarily on the presence of cardiac magnetic resonance (CMR) imaging identified fibrotic tissue as a key risk factor for predicting SCD in NICM patients. Evidence suggests that the extent of late gadolinium enhancement (LGE) on CMR, indicative of myocardial fibrosis, correlates with an increased risk of SCD, highlighting its potential role in refining risk stratification and guiding the decision-making process for ICD implantation in these patients [8, 9].

**Fig. 1:**
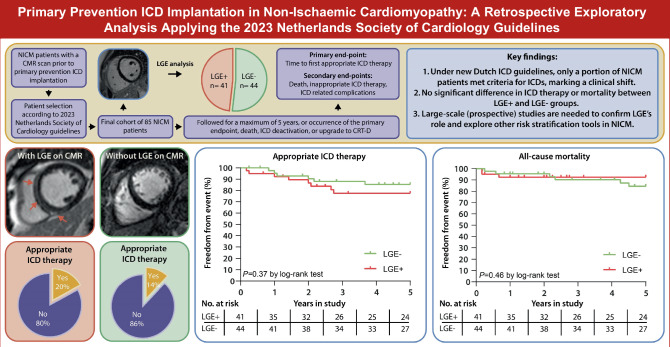
Infographic: Appropriate ICD therapy and all-cause mortality in NICM patients with and without LGE on CMR. Left-hand panels: An example of a patient demonstrating LGE on CMR (arrows) in a septal midwall and hinge-point pattern. Appropriate ICD therapy occurred in 20% of LGE+ patients during a 5-year follow-up period. Next to that, an example of a patient demonstrating no LGE on CMR. Appropriate ICD therapy occurred in 14% of LGE− patients during a 5-year follow-up period. Middle panel: Time-to-first-event curves for the occurrence of appropriate ICD therapy among NICM patients with (red) and without (green) LGE on CMR. Right-hand panel: Time-to-first-event curves for the occurrence of all-cause mortality among NICM patients with (red) and without (green) presence of LGE on CMR. *CMR* cardiac magnetic resonance, *ICD* implantable cardioverter-defibrillator, *LGE* late gadolinium enhancement, *NICM* non-ischaemic cardiomyopathy

**Fig. 2:**
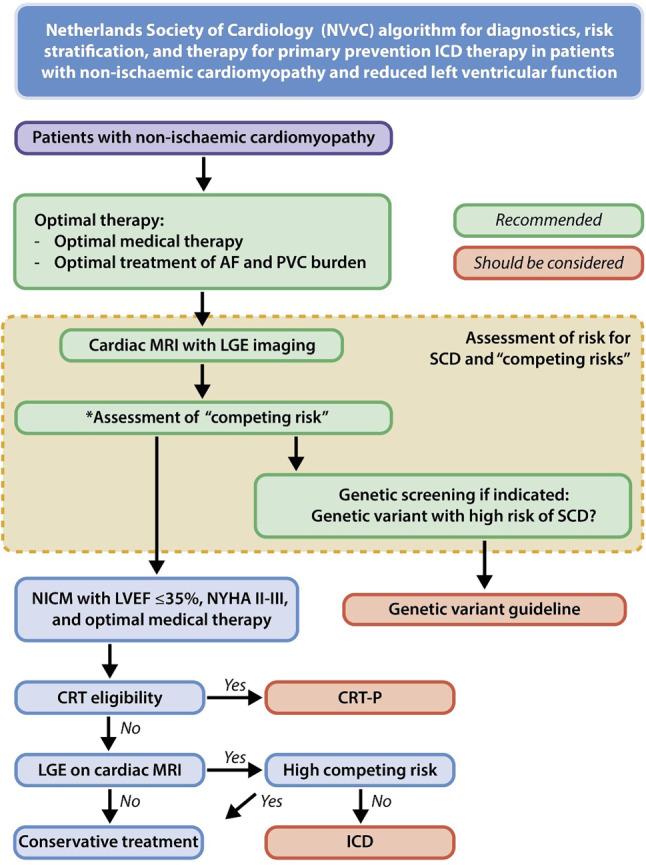
Netherlands Society of Cardiology (NVvC) algorithm for diagnostics, risk stratification, and therapy for primary prevention ICD therapy in patients with non-ischaemic cardiomyopathy and reduced left ventricular function. * based on the Heart Failure Meta-Score (http://www.hfmetascore.org). *AF* atrial fibrillation, *CMR* cardiac magnetic resonance, *CRT* cardiac resynchronisation therapy, *ICD* implantable cardioverter-defibrillator, *LGE* late gadolinium enhancement, *LV* left ventricle, *NICM* non-ischaemic cardiomyopathy, *PVC* premature ventricular contraction, *SCD* sudden cardiac death

In anticipation of these findings, a working group of the Netherlands Society of Cardiology (Nederlandse Vereniging voor Cardiologie, NVvC), in collaboration with the Dutch Healthcare Institute (Zorginstituut Nederland, ZiN), has reviewed and refined the European Society of Cardiology (ESC) guidelines [10]. These updated guidelines, now applicable in the Netherlands, recommend primary prevention ICD implantation only in NICM patients with LGE on CMR (Fig. 1). Additionally, patients with a specific genetic predisposition for SCD are treated according to specific risk models concerning these pathogenic variants, and patients with hypertrophic cardiomyopathy (HCM), inflammatory or infiltrative cardiomyopathy (e.g., cardiac amyloidosis or sarcoidosis) are treated according to ESC guidelines for ICD implantation (Fig. 2).

As there is a critical need to evaluate the impact of this more stringent Dutch interpretation of the ESC guidelines on patient outcomes, this exploratory single-centre study aims to retrospectively apply the 2023 national guidelines, including LGE assessment, to patients with NICM who have previously received an ICD according to the ESC guidelines.

## Methods

This is a retrospective, single-centre observational exploratory cohort study. The study was conducted in accordance with the Declaration of Helsinki. The collection and management of data were approved by the local medical ethics committee (Amsterdam University Medical Centre, Amsterdam, the Netherlands). The ethics board waived the requirement for informed consent, as the study involved a retrospective analysis of previously collected and anonymised data, posing at most minimal risk to participants. This waiver was granted in accordance with regulatory guidelines that stipulate the conditions under which informed consent can be ethically waived. The data underlying this article will be shared upon reasonable request to the corresponding author.

### Patient population

All consecutive NICM patients undergoing ICD implantation for the primary prevention of SCD at Amsterdam University Medical Centre, location VUMC, the Netherlands, between January 2009 and April 2022, in whom adequate-quality CMR imaging was available, were included in the study. The study population was further refined according to the 2023 position statement of the Task Force of the Netherlands Society of Cardiology [10]. Patients who had received CRT‑D were excluded.

Patients who carried specific genetic variants associated with an increased risk of SCD, such as the phospholamban (PLN) p.Arg14del mutation, lamin A/C (LMNA) mutation, filamin C (FLNC) mutation, receptor-binding motif (RBM20) mutation, and pathogenic desmoplakin (DSP) mutation, were excluded. Additionally, patients with hypertrophic cardiomyopathy (HCM), arrhythmogenic right ventricular cardiomyopathy (ARVC), Brugada syndrome, cardiac sarcoidosis, cardiac amyloidosis or congenital cardiac disease were excluded from the analysis.

### Patient follow-up and study endpoints

The primary endpoint of this study was the time to first appropriate ICD therapy, defined as antitachycardia pacing (ATP) and/or shock for the termination of ventricular arrhythmia. The secondary endpoint was all-cause mortality. Deaths were identified using the National Health and Social Care Information Service and the electronic medical record system.

Each patient was followed for a maximum of 5 years. This duration was selected as it aligns with established timelines for ICD studies [9]. Prolonged follow-up periods resulted in a decrease in patient numbers, partly due to the upgrade from ICD to CRT‑D in some patients. A 5-year follow-up duration allowed for adequate assessment of device efficacy and patient outcomes within a clinically relevant timeframe. A longer follow-up period has been examined and is available upon request to the corresponding author.

Routine device interrogations were performed at 10 days post-ICD implantation, 2 months post-implantation, and then every 6 months thereafter or in the event of device therapy (ATP or shock). The majority of patients were connected to remote monitoring systems, with event transmissions reviewed by specialised cardiac device technicians and electrophysiologists. ICD programming was based on standard clinical practice and was adjusted based on physicians’ discretion [11].

Patients were followed for the occurrence of the primary endpoint or death until 1 May 2024. Data from patients lost to follow-up were censored after their last clinical contact, as were data for patients undergoing ICD extraction, ICD deactivation, or upgrade to a CRT‑D.

### Inappropriate ICD therapy and ICD-related complications

Inappropriate ICD therapy and ICD-related complications were assessed throughout the follow-up period. Inappropriate ICD therapy was defined as device-delivered therapy (shock or ATP) triggered by non-ventricular arrhythmic causes, such as atrial fibrillation with rapid conduction, supraventricular tachycardia or lead noise. ICD-related complications were recorded and described for each individual case.

### CMR acquisition and CMR-LGE analysis

CMR scans were performed using either a 1.5 T or 3 T clinical MRI system (Siemens Avanto, Siemens Sonata, Siemens Sola, Siemens Vida, or GE Signa). LGE imaging included both long-axis and short-axis views to ensure a comprehensive evaluation of myocardial LGE distribution and extent.

Visual assessment of myocardial LGE was conducted by two independent readers. In cases of uncertainty regarding the presence and distribution of LGE, a third reviewer evaluated the LGE-CMR scan. The presence or absence of LGE was assessed for each myocardial segment using the American Heart Association (AHA) 17-segment model. The number of segments with myocardial LGE was recorded for each patient.

Additionally, myocardial LGE was categorised based on its distribution pattern, including midwall, epicardial, subendocardial, transmural, and hinge-point LGE. Midwall LGE was further classified into midwall stripe LGE and patchy midwall LGE. The localisation of LGE was identified as anterior, inferior, lateral, septal, or all-around.

### Statistical analysis

Patients with LGE were compared with patients without LGE on CMR (LGE+ group and LGE− group, respectively). T‑tests were performed on normally distributed continuous variables, while Mann-Whitney U tests were used for non-normally distributed continuous variables. For categorical variables, a chi-square test was employed.

For the time-to-event analyses, cumulative incidence curves were constructed using the Kaplan-Meier method, and hazard ratios with 95% confidence intervals were calculated using Cox proportional-hazards models. Additionally, a chi-square test was conducted to examine the relationship between the extent of LGE, the distribution pattern of left ventricular LGE, and the occurrence of appropriate ICD therapy and all-cause mortality.

Cox regression was used to identify parameters predicting the occurrence of appropriate ICD therapy. After performing the univariable analysis, multivariable regression was conducted using backward elimination. Predictive parameters with *p*-values < 0.1 were considered for the multivariable analysis.

Statistical analyses were performed using SPSS statistical software, version 28.

## Results

### Study population

**Fig. 3:**
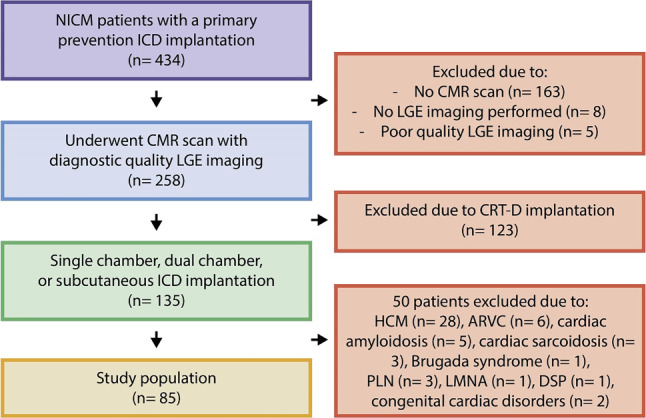
Flowchart illustrating the selection process of the study population. *ARVC* arrhythmogenic right ventricular cardiomyopathy, *CMR* cardiac magnetic resonance, *CRT‑D* cardiac resynchronisation therapy with defibrillator, *DSP* desmoplakin, *HCM* hypertrophic cardiomyopathy, *ICD* implantable cardioverter-defibrillator, *LGE* late gadolinium enhancement, *LMNA* lamin A/C, *NICM* non-ischaemic cardiomyopathy, *PLN* phospholamban

A cohort of 434 patients with NICM who had received an ICD for the primary prevention of SCD was identified; 263 of these patients had undergone LGE-CMR prior to implantation. After excluding 5 patients due to poor-quality LGE-CMR scans, 258 patients remained eligible for further analysis. Patients who had received a CRT‑D were then excluded (*n* *=* 123), as were those with HCM (*n* *=* 28), ARVC (*n* *=* 6), cardiac sarcoidosis (*n* *=* 3), cardiac amyloidosis (*n* *=* 5), Brugada syndrome (*n* *=* 1), and specific genetic variants associated with an increased SCD risk, including the PLN p.Arg14del mutation (*n* *=* 3), LMNA mutation (*n* *=* 1), and pathogenic DSP mutation (*n* *=* 1) (Fig. 3). Additionally, two patients with congenital cardiac disorders were excluded: one with Tetralogy of Fallot and another with Ebstein’s anomaly.

Following these exclusions, 85 patients remained for further analysis. Among these, 41 patients had LGE on CMR (LGE+ group, 48%), while 44 did not show LGE (LGE− group, 52%). Baseline characteristics of both groups are presented in Tab. 1, revealing no significant differences between the groups except for the presence of hypercholesterolaemia. The majority of patients (*n* *=* 81, 95%) received a transvenous device, while four patients (5%) received a subcutaneous ICD (S-ICD). The median follow-up duration was 5 years (interquartile range [IQR]: 2.0–5.0 years) for both the LGE+ group (5.0 years, IQR: 1.7–5.0 years) and the LGE− group (5.0 years, IQR: 2.2–5.0 years).

**Table 1:** Characteristics of the patients at baseline

	*LGE+* (*n* = 41)	*LGE−* (*n* = 44)	*p*-value
Mean age—years ± SD	64 ± 10	60 ± 12	0.20
Male sex—no. (%)	25 (61)	23 (52)	0.42
Hypertension—no. (%)	15 (47)	14 (45)	0.89
Diabetes—no. (%)	4 (10)	8 (20)	0.21
Hypercholesterolaemia—no. (%)	12 (38)	1 (4)	< 0.01
History of AF—no. (%)	9 (22)	10 (23)	0.93
Mean BMI—kg/m^2^	26.0 ± 4.3	26.4 ± 6.3	0.78
Mean LVEF—(%)	24.5 ± 8.9	26.4 ± 7.8	0.29
Mean QRS duration—msec	116 ± 26	116 ± 26	0.95
*Type ICD*			
– Single-chamber ICD—no. (%)	22 (54)	20 (45)	0.45
– Dual-chamber ICD—no. (%)	17 (41)	22 (50)	0.43
– Subcutaneous ICD—no. (%)	2 (5)	2 (5)	0.94
Amiodarone—no. (%)	5 (12)	1 (2)	0.07
Beta blocker—no. (%)	39 (95)	38 (86)	0.17
ACE or ATII antagonist—no. (%)	37 (90)	34 (77)	0.11
MRA (%)—no. (%)	19 (46)	15 (34)	0.25
Median time from CMR to device implantation (IQR)—months	2 (0–5)	2 (0–5)	> 0.99

*ACE* angiotensin-converting enzyme*, AF* atrial fibrillation*, ATII* angiotensin II*, BMI* body mass index*, CMR* cardiac magnetic resonance*, ICD* implantable cardioverter-defibrillator*, IQR* interquartile range*, LGE* late gadolinium enhancement, *LVEF* left ventricular ejection fraction*, MRA* mineralocorticoid receptor antagonist*, no.* number, *SD* standard deviation

### Appropriate ICD therapy

Among the 85 patients with ICDs included in the study, 14 (16%) experienced appropriate ICD therapy for ventricular arrhythmias. Of these 14 patients, 5 (36%) received ATP only, 3 (21%) received both ATP and shock, and 6 (43%) received shock therapy alone. The arrhythmia underlying the therapy was VF in two patients (14%) and VT in the remaining 12 patients (86%).

The primary endpoint of appropriate ICD therapy occurred in 8 patients in the LGE+ group and in 6 patients in the LGE− group, with a 5-year Kaplan-Meier estimated cumulative incidence of 20% and 14%, respectively (hazard ratio (HR) 1.62, confidence interval (CI) 0.56–4.63, *p* = 0.37) (Fig. 1).

In both univariate and multivariate analyses, no significant variables were found to be associated with the incidence of appropriate ICD therapy among NICM patients (Tab. 2).

**Table 2:** Univariable and multivariable backwards stepwise Cox regression analysis for parameters associated with appropriate ICD therapy

Dependent variable appropriate ICD therapy during follow-up	*Univariable*		*Multivariable*	
	HR (95% CI)	*p*-value	HR (95% CI)	*p*-value
Age (years)	0.99 (0.95–1.04)	0.67		
Sex (male)	0.55 (0.17–1.77)	0.32		
BMI (kg/m^2^)	0.96 (0.86–1.08)	0.48		
Single-chamber ICD	1.14 (0.46–4.15)	0.57		
Hypertension	0.54 (0.14–2.12)	0.38		
Hypercholesterolaemia	1.33 (0.34–5.16)	0.68		
Diabetes mellitus	2.01 (0.56–7.26)	0.29		
QRS duration (ms)	1.00 (0.98–1.02)	0.70		
LVEF (%)	0.97 (0.91–1.03)	0.35		
LGE on CMR	1.14 (0.40–3.30)	0.81		

*Predictors were considered for the multivariable analysis when p* *<* *0.1*

*BMI* body mass index*, CI* confidence interval*, CMR* cardiac magnetic resonance*, ICD* implantable cardioverter defibrillator*, LGE* late gadolinium enhancement*, LVEF* left ventricular ejection fraction

### All-cause mortality

Nine patients died over a 5-year follow-up period, indicating a 2% annual mortality rate. Among the LGE+ group, 3 patients died during follow-up, compared with 6 patients in the LGE− group (5-year Kaplan-Meier estimated cumulative incidence of 7% and 14%, respectively, HR 0.60 (95% CI 0.16–2.23), *p* = 0.46) (Fig. 1). The causes of death for the study population are summarised in Tab. S1 of the Electronic Supplementary Material. Among the 9 patients who died during the study period, 2 deaths (22%) were attributed to respiratory causes, 2 (22%) to heart failure and 2 (22%) to oncological causes. The cause of death was unknown in 3 patients (33%).

### Extent and distribution of LGE

The extent of LGE typically ranged from 1 to 4 segments (Fig. S1 in the Electronic Supplementary Material). Patients with LGE were divided into two groups based on the median number of segments with LGE: 1–2 segments and > 3 segments. No significant differences were observed between these groups in terms of appropriate ICD therapy (*p* = 0.37) or all-cause mortality (*p* = 0.41).

Additionally, the distribution pattern of LGE was assessed (Fig. S2 in the Electronic Supplementary Material). The midwall septal stripe and hinge-point LGE were the most common patterns, present in 56% and 51% of patients with LGE, respectively. Epicardial LGE was also frequent, occurring in 36% of cases. There was no significant association between appropriate ICD therapy or all-cause mortality and the presence of midwall septal stripe, hinge-point LGE, or epicardial LGE.

### Inappropriate ICD therapy and ICD-related complications

Inappropriate ICD therapy was observed in 9 patients (Tab. S2 in the Electronic Supplementary Material). The most common cause was supraventricular tachycardia, including atrial fibrillation, which accounted for 6 of the 9 cases (66%). Other causes of inappropriate therapy included lead fracture (*n* *=* 1/9, 11%), T‑wave oversensing (*n* *=* 1/9, 11%), and subcutaneous emphysema (*n* *=* 1/9, 11%).

Device-related complications occurred in 19 patients (22%) and are summarised in Tab. S3 in the Electronic Supplementary Material. The most frequent complications involved lead or device repositioning or replacement, occurring in 8 patients (9%). Other complications included pneumothorax in 4 patients (5%), pocket haematoma in 3 patients (4%), ICD-related infection in 1 patient (1%), a thrombotic event in 1 patient (1%), cardiac perforation in 1 patient (1%), and VF during lead positioning in 1 patient (1%).

## Discussion

In this single-centre retrospective exploratory cohort study of selected patients with NICM, approximately 50% exhibited LGE on CMR imaging. The patterns of LGE observed were consistent with non-ischaemic aetiologies, including septal midwall LGE, hinge-point LGE, and epicardial LGE. Over a 5-year follow-up period, 17% of patients received appropriate ICD therapy, while all-cause mortality was noted in 10% of the cohort. During follow-up, 11% of patients experienced inappropriate device therapy, while 22% encountered an ICD-related complication.

There was no significant difference in the rates of appropriate ICD therapy or all-cause mortality between patients with LGE (LGE+) and those without LGE (LGE−). The lack of significant prognostic differentiation between LGE+ and LGE− groups challenges the assumption that LGE alone should dictate the clinical decision-making process regarding ICD implantation in NICM patients. Notably, 14% of patients without LGE experienced appropriate ICD therapy. However, this study’s small sample size and limited statistical power are significant limitations, underscoring the need for a larger, multicentre study to validate these findings and provide more definitive answers. Further studies should also focus on identifying additional biomarkers or imaging features that may better predict outcomes in this patient population.

## Selection based on the 2023 national guidelines

Since 2023, the Netherlands has adopted more stringent guidelines for primary prevention ICD implantation in patients with NICM compared with the ESC guidelines (Fig. 2). Under the Dutch guidelines, patients who meet the criteria for CRT receive a CRT without a defibrillator function (i.e. a CRT-pacemaker). Additionally, patients with known pathogenic arrhythmogenic mutations, such as those associated with HCM or ARVC, are evaluated based on their specific SCD risk models and are considered for ICD implantation only if they have a high risk of SCD [12–14]. For the remaining NICM patients, the Dutch guidelines stipulate that the presence of LGE on CMR imaging is a necessary criterion for prophylactic ICD implantation [10].

In our cohort of 434 primary prevention NICM patients, 40% did not undergo a CMR scan prior to ICD implantation, thus preventing the assessment of LGE in a substantial portion of the patients. Historically, CMR imaging was not routinely conducted in patients diagnosed with NICM. However, recent guideline updates have increasingly recommended the use of CMR, significantly altering clinical practice [15].

Among the 258 patients who did undergo CMR, nearly half were found to be candidates for CRT, consistent with literature indicating that approximately half of NICM patients qualify for CRT due to significant ventricular conduction delay [16]. Moreover, in the remaining 135 patients who underwent CMR, more than one-third required further evaluation using specific risk models tailored to genetic variations or inflammatory/infiltrative heart diseases. The increased prevalence of genetic testing in recent years has likely contributed to a higher identification rate of individuals with arrhythmogenic mutations.

This trend towards more comprehensive diagnostic assessment, including both CMR and genetic testing, reflects a growing recognition of their importance in refining patient selection for ICD implantation. Among the remaining 85 NICM patients, most of whom had dilated cardiomyopathy of unknown aetiology, half displayed LGE on CMR imaging. Thus, within the group of 258 patients who underwent CMR scanning, only 35% (91/258 NICM patients) met the criteria for ICD implantation under the current, more stringent Dutch guidelines.

In contrast, under the broader ESC guidelines, which allow for ICD implantation in patients without LGE and also include those eligible for CRT‑D therapy, a significantly larger proportion of patients would qualify for ICD implantation.

### Appropriate ICD therapy and all-cause mortality in primary prevention NICM patients

In our study cohort, 17% of patients (14 out of 85) received appropriate ICD therapy over a 5-year follow-up period, corresponding to an annual incidence rate of 3%. This rate is relatively modest compared with rates reported in the literature. For instance, a recent pooled analysis of five primary prevention ICD trials, which included patients with both ischaemic cardiomyopathy and NICM, found a 6% annual incidence of appropriate ICD therapy in NICM patients [17]. The lower rate observed in our study may be attributed to the exclusion of NICM patients with an increased risk of ventricular arrhythmia based on specific underlying aetiologies.

In contrast, the more contemporary DO-IT (Dutch Outcome in ICD Therapy) study reported a 5.9% cumulative incidence of appropriate ICD therapy over 2 years in NICM patients, which aligns more closely with our findings. This suggests that our cohort, which excluded higher-risk patients, exhibited an incidence rate that is consistent with recent studies that have employed more stringent patient selection criteria [16].

Our study population also demonstrated a low incidence of all-cause mortality, with an annual rate of 2%. This contrasts with findings from the Danish Study to Assess the Efficacy of ICDs in Patients with Non-Ischaemic Systolic Heart Failure on Mortality (DANISH), which reported an annual mortality rate of 4%. The DANISH study, which involved over 1000 patients with NICM and an LVEF of ≤ 35%, found no significant mortality benefit from the use of ICD therapy compared with medical therapy alone [5]. The implications of the DANISH study are particularly relevant in light of our findings. Given the low incidence of both mortality and appropriate ICD therapy in NICM patients in our study cohort, the potential benefits of ICD implantation may be limited. This suggests that ICD therapy should be considered primarily for carefully selected NICM patients who are at an above-average risk for ventricular arrhythmia. The findings underscore the importance of thorough risk stratification prior to ICD implantation to avoid unnecessary procedures and reduce the risk of adverse events and complications associated with device implantation.

### LGE as a risk predictor for ventricular arrhythmias

Previous research has shown that the presence of LGE on CMR is a significant predictor of major ventricular arrhythmic events in patients with dilated NICM [9]. A recent meta-analysis including studies published between 2008 and 2022, with a median follow-up period of 3 years, reported a pooled odds ratio of 3.99 (95% CI 3.08–5.16), highlighting that LGE assessment may be a valuable parameter for primary prevention ICD implantation in NICM patients [18]. However, in our exploratory study, we found no significant association between the presence of LGE and appropriate ICD therapy, potentially due to the limited sample size and low event rate. Approximately half of the NICM patients in our study exhibited LGE on CMR, consistent with existing literature on LGE prevalence in this patient population [18]. Additionally, the prevalence of a midwall septal stripe in the LGE+ group aligned with current findings on LGE distribution in NICM patients [19]. Although specific LGE patterns such as midwall fibrosis have been linked to an increased risk of arrhythmias, our study did not observe a significant association between specific LGE distribution patterns and appropriate ICD therapy [20].

Therefore, our study does not provide conclusive evidence regarding the utility of LGE assessment as a primary risk stratification tool for ICD implantation in patients with NICM. Notably, 14% of patients without LGE on CMR still experienced appropriate ICD therapy, highlighting the necessity of closely monitoring patients who, under the new guidelines, do not receive an ICD. This finding also emphasises the need for large randomised trials to evaluate both the risk of SCD in NICM patients without LGE and the risk-benefit profile of prophylactic ICD implantation in those with LGE. Such studies are essential to establish more definitive guidelines for ICD therapy in this population. Furthermore, we encourage other centres to replicate our analysis to enable pooled data assessments, thereby allowing for a more comprehensive evaluation of arrhythmic risk and the role of LGE in NICM patients.

### Limitations

Our study has several limitations. First, in line with the retrospective and exploratory nature of this study on ICD therapy and all-cause mortality in NICM patients, the cohort size and event rate are modest. Second, LGE quantification was not performed because current quantification strategies are inconsistent and lack robust validation [21–23]. Moreover, the threshold for LGE extent and its associated risk remains undetermined [24]. Consequently, assessing the presence or absence of LGE visually was deemed practically reasonable, as endorsed by national guidelines for ICD implantation in NICM patients. Thirdly, current guidelines recommend genetic testing for diagnosis and risk stratification in NICM [25]. However, this was not routinely performed in patients included in our analysis. Fourthly, CRT patients were excluded to ensure a homogeneous cohort, as CRT is a heart failure therapy that independently modifies arrhythmic risk and mortality, potentially confounding the analysis of ICD therapy events [26, 27]. Future studies should focus specifically on the CRT‑D population. Finally, heart failure therapy has significantly improved over the years, including the standard use of SGLT2 inhibitors and angiotensin receptor-neprilysin inhibitors, which significantly improve outcomes in heart failure patients [28]. This may have influenced the results of this study significantly.

## Conclusion

Although CMR-determined LGE was observed in approximately half of the patients with NICM, our study found no significant differences in the rates of appropriate ICD therapy or all-cause mortality between patients with and without LGE over a 5-year follow-up period. However, it is important to underscore that this was a single-centre, exploratory, retrospective study with a limited sample size and, as such, the statistical power was insufficient to draw definitive conclusions.

This study is intended as an initial step for future research and highlights the need for larger, multicentre retrospective and prospective investigations to further elucidate the prognostic value of LGE in NICM. We call on other institutions to collaborate in advancing this important area of study.

## Supplementary Information

Fig. S1 Left ventricular LGE extent in the study population. Upper part: Schematic representation of the AHA 17 segments of the LV, accompanied by CMR images illustrating the basal, mid-cavity, and apical segments. Lower part: A bar chart displaying the percentage of patients with LGE in the amount of AHA segments. AHA: American Heart Association, CMR: cardiac magnetic resonance, LGE: late gadolinium enhancement, LV: left ventricle, NICM: non-ischemic cardiomyopathy

Fig. S2 Left ventricular LGE distribution pattern in the study population. Upper part: Schematic illustration of the various LV LGE distribution patterns, highlighting potential areas of enhancement (anterior, inferior, lateral, septal, and global). Lower part: Bar chart depicting the percentage of patients exhibiting LGE in each distribution pattern. CMR: cardiac magnetic resonance, LGE: late gadolinium enhancement, LV: left ventricle, NICM: non-ischemic cardiomyopathy

Table S1 [s. MS_9]

Table S2 [s. MS_10]

Table S3 [s. MS_11]
