# Supplementary figures and images for: Prognostic value of late gadolinium enhancement cardiac MRI for ICD therapy in non-ischaemic cardiomyopathy: A 5-year cohort study
Source: Neth Heart J. 2025 Mar 25;33(5):163–71. doi: 10.1007/s12471-025-01946-3 (PMC12014978; doi:10.1007/s12471-025-01946-3)

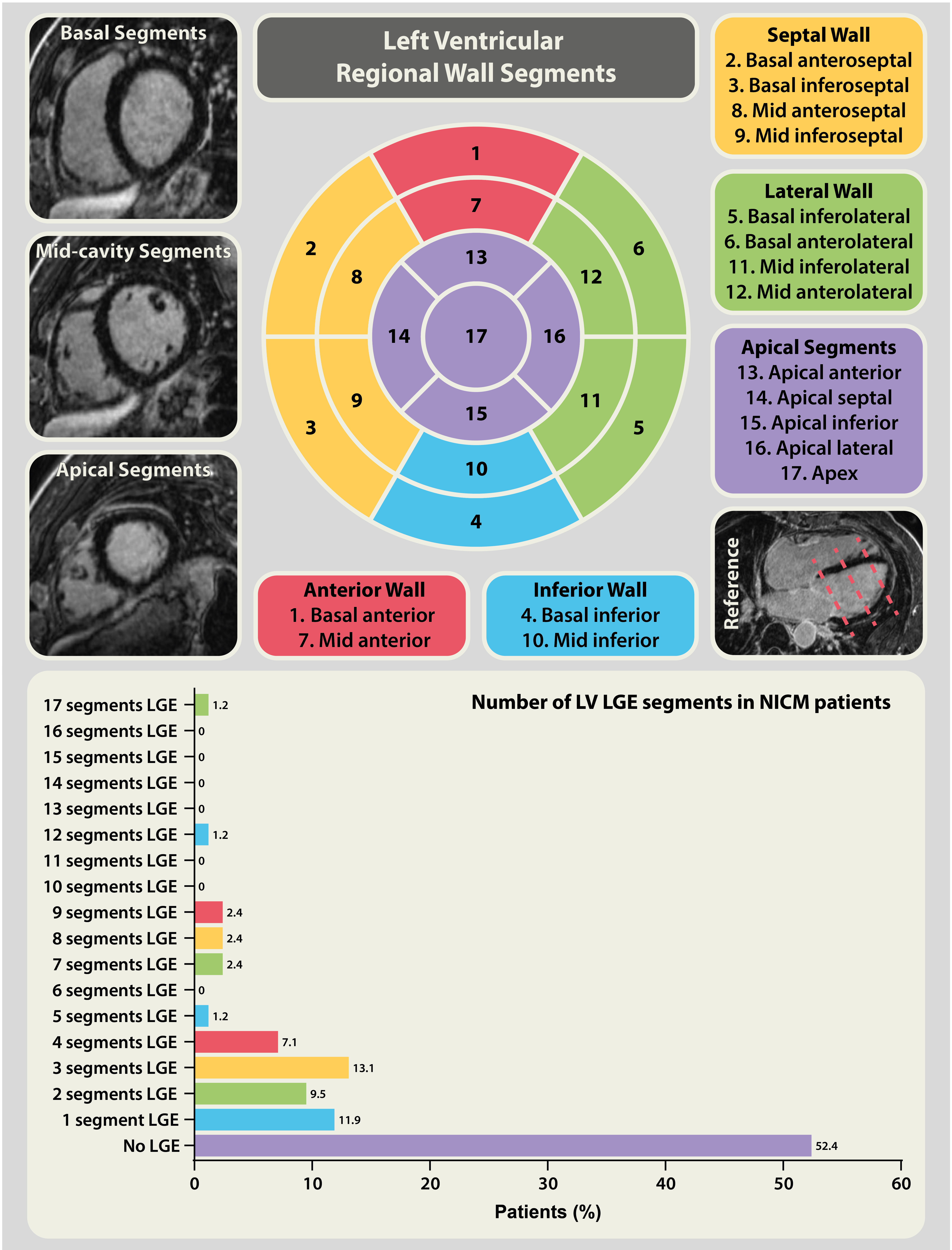

Supplement: Supplementary file 1 — Fig. S1 Left ventricular LGE extent in the study population. Upper part: Schematic representation of the AHA 17 segments of the LV, accompanied by CMR images illustrating the basal, mid-cavity, and apical segments. Lower part: A bar chart displaying the percentage of patients with LGE in the amount of AHA segments. AHA: American Heart Association, CMR: cardiac magnetic resonance, LGE: late gadolinium enhancement, LV: left ventricle, NICM: non-ischemic cardiomyopathy [file 12471_2025_1946_MOESM1_ESM.jpg]

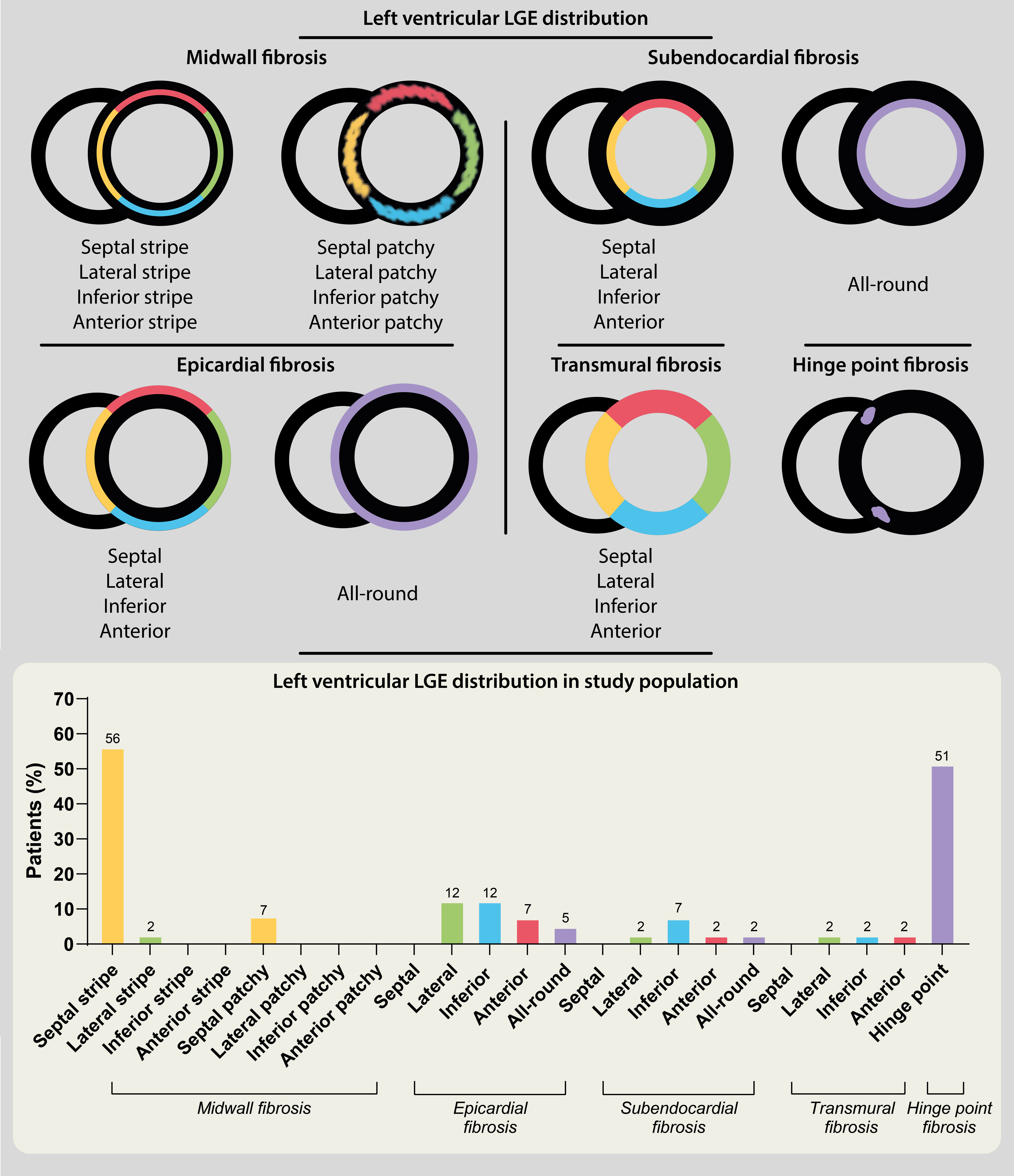

Supplement: Supplementary file 2 — Fig. S2 Left ventricular LGE distribution pattern in the study population. Upper part: Schematic illustration of the various LV LGE distribution patterns, highlighting potential areas of enhancement (anterior, inferior, lateral, septal, and global). Lower part: Bar chart depicting the percentage of patients exhibiting LGE in each distribution pattern. CMR: cardiac magnetic resonance, LGE: late gadolinium enhancement, LV: left ventricle, NICM: non-ischemic cardiomyopathy [file 12471_2025_1946_MOESM2_ESM.jpg]
