# Supplementary material for: Prognostic value of late gadolinium enhancement cardiac MRI for ICD therapy in non-ischaemic cardiomyopathy: A 5-year cohort study
Source: Neth Heart J. 2025 Mar 25;33(5):163–71. doi: 10.1007/s12471-025-01946-3 (PMC12014978; doi:10.1007/s12471-025-01946-3)
Supplement: Supplementary file 3 — Table S1 [s. MS_9] [file 12471_2025_1946_MOESM3_ESM.docx]

| **Table S1:** Cause of death, *n.* | |
| --- | --- |
| Respiratory cause | 2/9 (22%) |
| Heart failure | 2/9 (22%) |
| Oncological cause | 2/9 (22%) |
| Unknown | 3/9 (33%) |
