# Supplementary material for: Prognostic value of late gadolinium enhancement cardiac MRI for ICD therapy in non-ischaemic cardiomyopathy: A 5-year cohort study
Source: Neth Heart J. 2025 Mar 25;33(5):163–71. doi: 10.1007/s12471-025-01946-3 (PMC12014978; doi:10.1007/s12471-025-01946-3)
Supplement: Supplementary file 4 — Table S2 [s. MS_10] [file 12471_2025_1946_MOESM4_ESM.docx]

| Table S2: Reasons of inappropriate ICD therapy, *n.* | | |
| --- | --- | --- |
| Supraventricular tachycardia (including AF) | 6/9 (66%) | |
| Lead fracture | 1/9 (11%) |  |
| T-wave oversensing | 1/9 (11%) |  |
| Subcutaneous emphysema | 1/9 (11%) |  |
| *AF: atrial fibrillation, ICD: implantable cardioverter defibrillator.* | | |
