# Supplementary material for: Prognostic value of late gadolinium enhancement cardiac MRI for ICD therapy in non-ischaemic cardiomyopathy: A 5-year cohort study
Source: Neth Heart J. 2025 Mar 25;33(5):163–71. doi: 10.1007/s12471-025-01946-3 (PMC12014978; doi:10.1007/s12471-025-01946-3)
Supplement: Supplementary file 5 — Table S3 [s. MS_11] [file 12471_2025_1946_MOESM5_ESM.docx]

| **Table S3:** Device-related complications, *n.* |  |
| --- | --- |
| Lead or device related complication resulting in repositioning or replacement | 8 (9%) |
| Pneumothorax | 4 (5%) |
| Pocket hematoma | 3 (4%) |
| ICD-related infection | 1 (1%) |
| Thrombotic event | 1 (1%) |
| Cardiac perforation | 1 (1%) |
| Other* | 1 (1%) |
| Total complications | 19 (22%) |
| **VF due to lead positioning during implantation*  *ICD: implantable cardioverter-defibrillator* |  |
